# An e-health transition intervention for youth with brain-based disabilities: Pilot and feasibility results from a Randomized Controlled Trial

**DOI:** 10.1016/j.hctj.2026.100144

**Published:** 2026-06-10

**Authors:** Jan Willem Gorter, Ronen Rozenblum, Adrienne H. Kovacs, Khush H. Amaria, Lehana Thabane, Barb Galuppi, Sonya Strohm, Linda Nguyen, Alicia Via-Dufresne Ley, Hana Alazem, John Andersen, Rima Azar, Kerry Boyd, Caitlin Cassidy, C.J. Curran, Claire Dawe-McCord, Shelley Doucet, Anne Fournier, Michael Frost, Dilshad Kassam-Lallani, Alison Luke, Anna McCormick, JoAnne Mosel, Connie Putterman, Michael Shevell, Kathy N. Speechley, Donna Thomson, Lonnie Zwaigenbaum, Ariane Marelli, CHILD-BRIGHT READYorNotTM Brain-Based Disabilities Trial Study Group

**Affiliations:** a CanChild Centre for Childhood Disability Research, McMaster University, Hamilton, ON, Canada; b Department of Pediatrics, McMaster University, Hamilton, ON, Canada; c Department of Rehabilitation, Physical Therapy Science and Sports, UMC Utrecht Brain Center, University Medical Center Utrecht, Utrecht, the Netherlands; d Centre of Excellence for Rehabilitation Medicine, UMC Utrecht Brain Center, University Medical Center Utrecht and De Hoogstraat Rehabilitation, Utrecht, the Netherlands; e Brigham and Women's Hospital and Harvard Medical School, Boston, MA, United States; f Equilibria Psychological Health, Toronto, ON, Canada; g CBT Associates, A CloudMD Company, Toronto, ON, Canada; h Department of Health Research Methods, Evidence, and Impact, McMaster University, Hamilton, ON, Canada; i Biostatistics Unit, St. Joseph's Healthcare Hamilton, Hamilton, ON, Canada; j Cardiovascular Health Across the Lifespan Program, Research Institute of the McGill University Health Centre, Montreal, QC, Canada; k University of Ottawa, Ottawa, ON, Canada; l Division of Developmental Medicine and Rehabilitation, CHEO, Ottawa, ON, Canada; m Department of Pediatrics, University of Alberta, Edmonton, AB, Canada; n Psychology Department, Mount Allison University, Sackville, New Brunswick, Canada; o Department of Psychiatry & Behavioral Neurosciences, McMaster University, Hamilton, ON, Canada; p Parkwood Institute, St Joseph's Health Care London, Parkwood Institute Research, London, Canada; q Department of Physical Medicine and Rehabilitation, Western University, London, Canada; r Holland Bloorview Kids Rehabilitation Hospital, Toronto, ON, Canada; s Cumming School of Medicine, University of Calgary, Calgary, AB, Canada; t Patient and Family Partner, CHILD-BRIGHT READYorNotTM Brain-Based Disabilities Project, Canada; u Faculty of Nursing & Health Sciences, University of New Brunswick Saint John, Saint John, NB, Canada; v Centre for Research in Integrated Care, University of New Brunswick Saint John, Saint John, NB, Canada; w Département de Pédiatrie, Faculté de Médecine, Université de Montréal, Montreal, QC, Canada; x Département de Pédiatrie, Centre Hospitalier Universitaire Sainte-Justine, Montreal, QC, Canada; y Spina Bifida and Spinal Cord Injury Program, Holland Bloorview Kids Rehabilitation Hospital, ON, Canada; z CHEO Research Institute, CHEO, Ottawa, Canada; aa Research Institute-McGill University Health Centre, Montreal, QC, Canada; ab Departments of Pediatrics and Neurology/Neurosurgery, McGill University, Montreal, QC, Canada; ac Department of Epidemiology and Biostatistics, Western University, London, ON, Canada; ad Child Health Research Institute, Lawson Health Research Institute, London, ON, Canada; ae Department of Paediatrics, Western University, London, ON, Canada; af Department of Pediatrics, Faculty of Medicine & Dentistry, University of Alberta, Edmonton, AB, Canada; ag McGill Adult Unit for Congenital Heart Disease Excellence, McGill University Health Centre, Montreal, QC, Canada; ah Cardiovascular Health Across the Lifespan Program, Research Institute of the McGill University Health Centre, Montreal, QC, Canada

**Keywords:** Adolescents, Healthcare transition, E-health, Disabilities, Self-management, Transition readiness, Patient engagement

## Abstract

**Background:**

Youth with brain-based disabilities (BBD) need tailored support when preparing for adult healthcare. We originally designed a full randomized controlled trial (RCT) to test whether the MyREADY Transition™ BBD App improved transition readiness among youth aged 15–17 years with autism spectrum disorder, cerebral palsy, epilepsy, fetal alcohol spectrum disorder, or spina bifida. Due to slow recruitment, the full RCT was halted and pivoted to a stand-alone pilot and feasibility trial.

**Methods:**

This mixed method, patient-oriented pilot RCT (2019–2022, Canada) evaluated process, resource, management, and scientific feasibility, along with engagement with the App. Scientific feasibility focused on self-management outcomes (TRAQ and Transition-Q) among 43 youth (mean age 15.9 ± 0.8; 19 intervention, 24 control). Interviews and surveys provided additional perspectives on feasibility and user experience.

**Results:**

Recruitment achieved only 43 of the planned 264 participants, and App engagement was modest. Almost all intervention participants logged into the App at least once, but on average completed less than one third of the curriculum. Qualitative findings suggested the App’s content was relevant and useful. Participants, caregivers, and healthcare providers emphasized the need for tailored, collaborative approaches to transition preparation.

**Conclusion:**

Challenges included slow recruitment and limited sustained use of an e health intervention among youth with BBD. Despite the pivot, the trial generated pragmatic insights for future transition research. Youth and parent recommendations underscored the importance of customizable content and strategies that enhance motivation and engagement. Additional pilot work is needed to refine and optimize digital transition supports.

## Highlights

• The importance of multiple recruitment strategies (e.g., community and online recruitment, building personal connections).
• Youth motivation (along the continuum of contemplation, preparation, and action stages of change) plays a role in the success of healthcare transition strategies.
• Future research should plan and implement motivational strategies.

## Graphical Abstract

**Figure:**
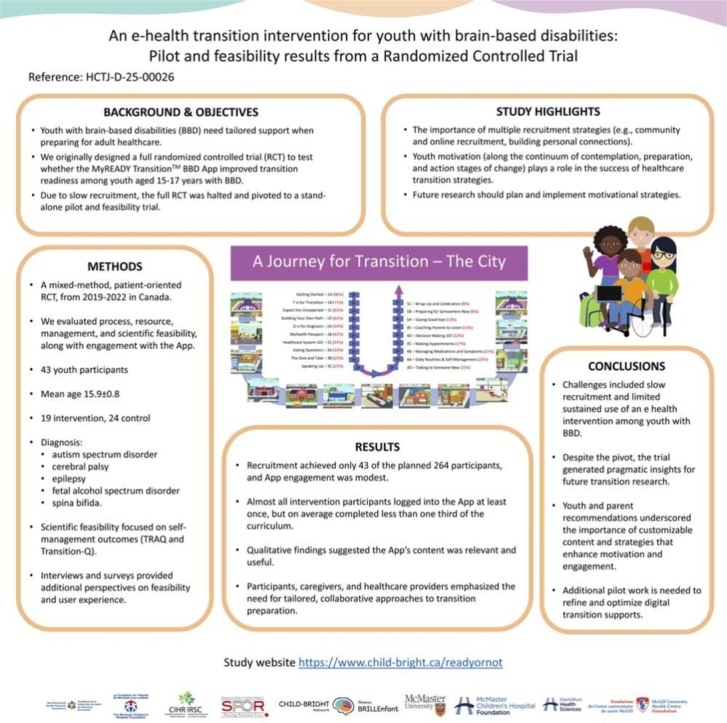

## 1 Introduction

Although most youth with neurodevelopmental brain-based disabilities (BBD) are diagnosed in childhood, youth with these conditions continue to have healthcare needs as they reach adulthood. Poor healthcare transition can have negative health outcomes and can result in poor quality of life for youth with BBDs, such as autism spectrum disorder, cerebral palsy, epilepsy, fetal alcohol spectrum disorder, and spina bifida.^1^ Thus, there is a need for proactive strategies to educate, empower, and support youth as they transition from pediatric to adult healthcare.^2^ There have been consistent calls for developing interventions and youth-centered resources with health literacy and healthcare self-management as critical developmental milestones.^3^

Health information technology (HIT), such as e-health applications (“Apps”), may be an appealing, accessible, and flexible way to engage youth. Given the unique challenges faced by youth with BBD, we evaluated existing BBD transition tools and developed the MyREADY Transition™ BBD App with the goal of improving the transition readiness of youth.^4^ The App was developed with researchers, healthcare professionals, technology experts, youth, and families working in partnership.^5^ Our App was designed for a range of brain-based disabilities, acknowledging that while they have different underlying health conditions they share common needs and challenges in acquiring health literacy (i.e. autonomy and learning to navigate the health care).^3^ The App consists of 19 sessions organized into five ordered sections; the second section was tailored to include an individualized and diagnosis-specific review of their condition, what it means, and understanding what one can do to take charge.

Evaluation of HIT is a continuous iterative process, including assessment of prototypes,^6^ and there is value in reporting on tests of program feasibility and initial effects.^7^, ^8^ We designed a randomized controlled trial (RCT) to investigate the App in a “real-world” setting. We hypothesized that youth with BBD aged 15–17 years who receive the patient-facing MyREADY Transition™ BBD App would report improved transition readiness at 6-month follow-up compared with usual care alone.

**Fig. 1:**
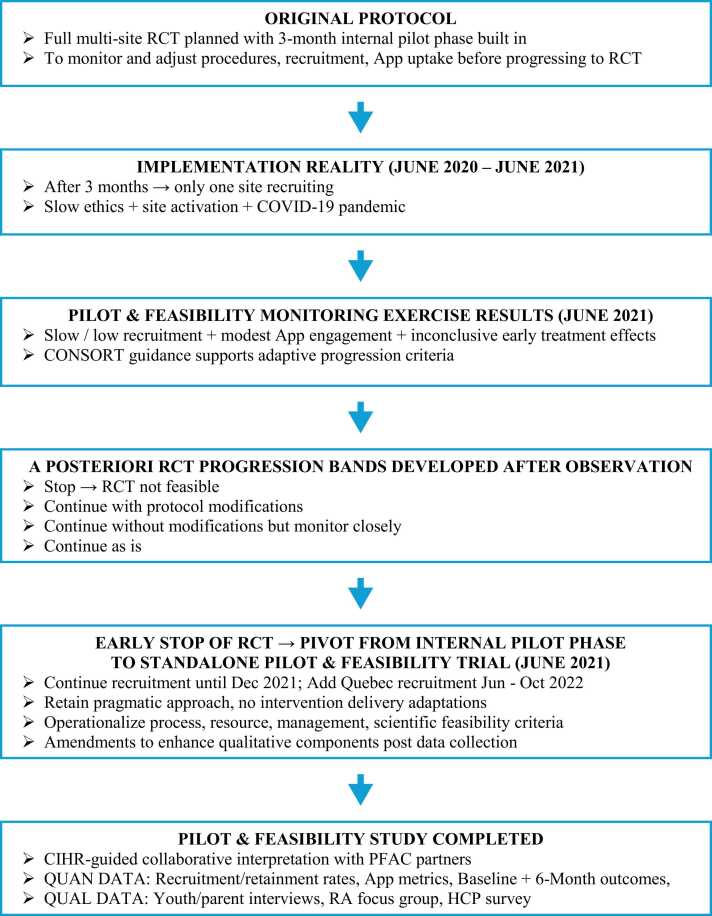
Pivot from Original Full RCT to Standalone Pilot and Feasibility Trial.

Due to slow recruitment, the trial was halted prematurely, and we pivoted the original full RCT to a stand-alone pilot and feasibility trial (Fig. 1). Herein, we describe our lessons learned and formal evaluation using pre-determined criteria for success, within an established feasibility framework.^7^ We report information about process, resource, management, and scientific feasibility as well as engagement with our App intervention designed to empower youth with BBD in their healthcare transition readiness. For scientific feasibility of pilot studies, researchers have an ethical and scientific obligation to attempt publishing the results of every research endeavor.^7^ The focus should be on feasibility goals rather than statistical significance; thus, we report estimates of initial treatment effects even though pilot studies typically are not powered to show statistically significant results. This aligns with FAIR Data Principles, which state that research data should be Findable, Accessible, Interoperable, and Reusable.^9^

## 2 Methods

### 2.1 Study design

This study employed a two-group RCT design with repeated measures at two time points. The protocols for our internal pilot phase and our full-scale, mixed methods, patient-oriented RCT were published in 2021.^1^ Ethics approval was obtained at the nine Canadian clinical sites in Ontario, Alberta, the Maritimes (New Brunswick and Nova Scotia), and Quebec. In addition to the traditional recruitment approach of having a clinical point of contact at each site, we incorporated online recruitment through social media as described in the protocol.^1^ We designed our study in collaboration with patients, families, and healthcare professionals. We partnered with a Patient and Family Advisory Council (PFAC) throughout the trial to enhance participant recruitment, data collection, engagement planning, and knowledge translation.^5^ By 2023, 14 partners (eight young adults and six parents) had actively contributed to the PFAC throughout the project, spanning six years. Fig. 1 describes the design pivot from original full RCT to a stand-alone pilot and feasibility trial.

### 2.2 Participants

#### 2.2.1 Recruitment and eligibility

RCT participants were recruited online through social media and from clinical sites. Study inclusion criteria were: 1) age 15–17 years; 2) a diagnosis of autism spectrum disorder, cerebral palsy, epilepsy, fetal alcohol spectrum disorder or spina bifida; 3) receiving pediatric care in one of the four study regions and for whom a discharge from pediatric care was planned in ≥ 6 months; 4) cognitive ability to provide informed assent and the ability to read and understand English or French; 5) personal access to the internet and an available smartphone, iPad/tablet or desktop computer; and 6) TRANSITION-Q^10^ score of > 40 (as a screen to confirm a minimum threshold for transition readiness). TRANSITION-Q is a 14-item transition readiness/self-management ability scale. All study recruitment (between June 2020 and October 2022) occurred in the context of the COVID−19 pandemic, which was declared on March 11, 2020, by the World Health Organization.

#### 2.2.2 Consent process

Prior to data collection, all youth provided assent, and a parent/caregiver provided consent both for their child and for themselves. To accommodate physical distancing measures as per COVID−19 mandates, either verbal or written consent procedures were offered. Parent/caregiver participants reported the sex assigned on their children’s birth certificates. Youth reported the gender with which they self-identified (e.g., girl/young woman, boy/young man, transgender person, other [e.g., non-binary]).

### 2.3 Randomization and masking

Randomization was stratified by region with a 1:1 allocation ratio for patients into either the intervention or control group. The unit of randomization was the patient. We used variable block randomization with block sizes of 2, 4, 6 and 8. Allocation was completed via REDCap (Research Electronic Data Capture) using a centralized allocation system generated by the Biostatistics Unit at St. Joseph’s Hospital in Hamilton, Ontario, Canada.

Due to the nature of the intervention, it was not possible to mask participants to group allocation, however, outcome assessment and data analysis were blinded. Participants in the control group were aware that this study was investigating an App but were not given detailed information about its content or design.

### 2.4 Procedures

#### 2.4.1 Study intervention (intervention group)

The MyREADY Transition™ BBD App uses a “Journey in the City” concept to represent the journey in transition to adult care. A mentor (virtual coach) helps users navigate through different buildings and locations in the App’s “city,” each of which sequentially introduces educational topics pertaining to the transition to adult care. The App consists of 19 sessions organized into five ordered sections comprising 5–7 h of total content. The recommended exposure to the App intervention was between one session per day (which would result in completion within 19 days) and one session per week (completion within 19 weeks). Additional games and fun activities were incorporated to encourage youth to explore the App between official educational sessions.^4^

During the baseline study visit, a research assistant (RA) supported participants in the intervention group to download the App on their device. Together, they watched an introductory video to familiarize youth with the App’s first session. To optimize exposure and uptake of the App during the trial, the RA also shared a patient-facing handout (Supplemental File 1), a technical support website with tutorial videos and frequently asked questions, and standardized encouragement messages. Research staff were available to troubleshoot technical support issues.

Healthcare providers and parents/caregivers of youth in the intervention group were also given a handout explaining that the researchers wanted to study how youth were using the App independently and offering suggestions about ways to support youth without influencing their use of the App (Supplemental File 2 and 3).

#### 2.4.2 Usual Care (intervention and control groups)

Participants randomized to the control group continued with the usual care they had already been receiving. Youth in both the control and intervention groups, along with their parent/caregiver, were given a standard handout providing a basic overview of what they might expect as they prepared for transition.^1^ This was to ensure all study participants had a minimum standard of transition preparation beyond their usual care.

### 2.5 Data collection

Quantitative and qualitative data were collected, with all data collection forms available in English and French. Detailed demographic information was collected at baseline for descriptive analysis of the study sample. Youth and parent/caregiver quantitative outcome measures were administered at baseline and 6 months (specific measures described below). Parents/caregivers were asked to report any adverse events during the study. App access and exposure were assessed quantitatively using the App’s Content Management System. As part of the embedded qualitative study, all English-speaking youth and parents/caregivers in the intervention group were invited to participate in a one-on-one, semi-structured interview using interview guides developed together with our PFAC partners. Interviews were conducted over Zoom videoconferencing^11^ after the 6-month study visit. The interview questions complemented our quantitative findings related to the App by capturing process, user experience and satisfaction with the App and its features, as well as opportunities to improve the App (Supplemental File 4 and 5).

After data collection, ethics approval was obtained for a focus group with the RA staff, facilitated by a graduate trainee (LN). The purpose was to enhance our feasibility exploration and reporting by gathering qualitative information about RA experiences related to the processes, resources, and management of the study, as well as the impact of the COVID−19 pandemic. Synchronous/asynchronous feedback was received from six RAs, representing all RCT regions (Supplemental File 6).

To further enhance our feasibility exploration and reporting, we conducted an electronic survey of healthcare providers (HCPs) after the trial ended. The 19 participants invited to complete the survey were HCPs involved in recruiting participants in all four regions (11 in Ontario, 3 in Alberta, 3 in the Maritimes, and 2 in Quebec). The survey included two open-ended questions to learn about HCP experience recruiting youth during the COVID−19 pandemic and their perspective on integrating the App into practice. Responses were received from five HCPs, representing one study region only (Ontario) (Supplemental File 7).

We embraced guidance from the Canadian Institutes of Health Research (CIHR)^12^ to interpret study data in collaboration with all partners. Patients, families, researchers, recruitment site investigators, and research staff were encouraged to add their perspectives during our collaborative interpretation meetings which were recorded and shared to allow for asynchronous participation.

### 2.6 Outcomes

In this study we evaluated process, resource, management and scientific feasibility as well as engagement with the App intervention.

#### 2.6.1 Feasibility: process, resource, management, and scientific

Table 1 describes the criteria for success and method of analysis for each of the feasibility outcomes: process (recruitment rate, engagement with the App), resource (retention rate and intervention uptake), management (ethics approval time), and scientific (initial treatment effects on RCT outcomes).

**Table 1:** Feasibility Framework with key feasibility outcomes, criteria for success, method of analysis and results.

Outcome	Criteria for success	Method of analysis	Results
**PROCESS FEASIBILITY:**			
Recruitment rate	Recruit 4 participants per month per region	Descriptive mean per month (95% Confidence Interval [CI])	Criterion not met Ontario n = 29 over 19 months. Mean= 1.5 (−1.3, 4.4) Alberta n = 10 over 13 months. Mean= 0.8 (−0.7, 2.2) Maritimes n = 7 over 10 months. Mean= 0.7 (−1.8, 3.2) Quebec n = 6 over 5 months. Mean= 1.2 (−1.4, 3.8)
App access	N/A	Average number of times an intervention participant logged into the App	Mean (SD)= 11.2 (9.5) n = 24
App exposure	N/A	Percentage of participants in the intervention group who completed each session in the App	See Fig. 3
App usability	> 70 System Usability Scale (SUS) score indicates acceptability	Perceived value, experience, and satisfaction with the intervention on the system usability scale	Criterion not met Mean= 65.4 SD= 26.8
**RESOURCE FEASIBILITY:**			
Retention rate	> 90% of participants complete all study visits	Descriptive proportion (95% Confidence Interval [CI])	Criterion not met 43/52 = 82.7% (0.7, 0.9)
Uptake of intervention	> 90% of intervention participants download App on their device and complete orientation	Descriptive proportion (95% Confidence Interval [CI])	Criterion met 23/24 = 95.8% (0.8, 1.0)
**MANAGEMENT FEASIBILITY:**			
Ethics approval time	Total number of days from submission to initial approval notiﬁcation	N/A	Total days for provincial and secondary site approval: Mean= 245.8 days Median= 210 days Range= 64–496 days n = 9 sites Total days for secondary streamlined site approval only: Mean= 60.4 days Median= 43 days Range= 4–125 days n = 5 sites
**SCIENTIFIC FEASIBILITY**			
Initial Treatment effects	N/A	Estimated effects of intervention on quantitative RCT outcomes at 6-month follow up	Described in Results and in Supplemental File 8.

#### 2.6.2 Engagement with the App

Engagement with the App was assessed in terms of access, exposure, and usability. Access and exposure were assessed quantitatively using the App’s Content Management System which collected registration and device information (i.e., device type and operating system), user login times and dates, and App session completion. Youth in the intervention group also completed the System Usability Scale^13^ at the 6-month study visit, using a Likert scale to indicate level of agreement with a series of ten statements. This provided a quantitative assessment of the App and its features, its perceived value, and participants’ overall experience and satisfaction with the App. The interviews with youth and parents/caregivers in the intervention group complemented our quantitative findings related to App engagement.

#### 2.6.3 Scientific feasibility

To report the scientific feasibility of the full-scale RCT, we included all data collected at baseline and at follow-up as part of the pilot RCT.

The full RCT primary outcome was transition readiness, measured with the 29-item version of the TRAQ,^14^ which includes a self-management domain (16 items) and a self-advocacy domain (13 items). Each item is scored from 1 to 5, where 1 = ‘I do not need to do this’, 2 = ‘I do not know how but I want to learn’, 3 = ‘I am learning to do this’, 4 = ‘I have started doing this’ and 5 = ‘I always do this when I need to’. The TRAQ is a validated, patient-centered measure of transition readiness^14^ and is designed to be self-administered at 6-month intervals.

A variety of other clinical outcomes were assessed. TRANSITION-Q^10^ was also used as a secondary measure of transition readiness. The Canadian Occupational Performance Measure (COPM)^15^ was used to identify self-care, productivity and leisure goals that were most important to study participants. Youth identified up to five goals at baseline and rated their performance and satisfaction on a 10-point scale at baseline and 6-months. The PedsQL™ Pediatric Quality of Life Instrument, Generic Core and Teen Report (13–18 years)^16^ was used to evaluate any impact on quality of life during the study. To assess the impact of the App on families’ healthcare experiences, parents/caregivers completed the Measure of Process of Care (family-centered care).^17^ Youth completed the Newest Vital Sign^18^ health literacy measure at baseline. Details about original RCT outcome measures are provided in our published protocol.^1^

### 2.7 Statistical Analysis

Our mixed methods approach combined both quantitative and qualitative research methods and techniques, a widespread practice in patient-centered research, to help answer complex research questions and allow for stronger and richer evidence than could be accomplished by a single method alone.

#### 2.7.1 Quantitative analysis

Patient demographic data and feasibility measures were reported using descriptive statistics including means and standard deviations for normally distributed data and counts and percentages for categorical data. To assess scientific feasibility (RCT outcomes) quantitative analyses were conducted separately to compare the intervention and usual control groups. The analyses were intention-to-treat; multiple imputation was used to handle missing data. All analyses were performed using R software Version 4.2.1.^19^ To determine the treatment effect, an analysis of covariance (ANCOVA) was performed. All analyses were adjusted for baseline and the effect of region was modelled as a fixed effect. As there were no interaction effects observed, all interactions terms were dropped in the final model. The treatment effect sizes from the analyses were reported as mean group differences accompanied by their corresponding 95% confidence intervals (CIs) and p values; alpha was set at 0.05 (two-sided).

#### 2.7.2 Qualitative analysis

Qualitative description informed data collection and analyses for the embedded qualitative study.^20^ This form of naturalistic inquiry is suited to mixed method designs, and studies where accounts are required directly from those who have experienced a phenomenon.^20^ All interview recordings were transcribed verbatim with identifying information removed. Data were stored and managed electronically using NVivo 11. A rapid qualitative analysis approach^21^ was used to synthesize the qualitative interview data as well as to triangulate findings with quantitative data for all RCT aims. This action-oriented approach to qualitative data analysis involves using a template to extract relevant concepts from individual transcripts followed by matrix analyses, which allows for mapping of variation across respondents. Summary templates were developed collaboratively by two research staff (BG and SS) who piloted their suitability for extracting and synthesizing data for the specific aims and goals of this project prior to their implementation.

Due to the intervention being short term with low risk to participant safety, we did not have a formal Data Safety Monitoring Board. The trial is registered with clinicaltrials.gov (NCT03852550).

### 2.8 Role of the funding source

The funder of the study had no role in study design, data collection, data analysis, data interpretation, or writing of the report.

## 3 Results

### 3.1 Participants

**Fig. 2:**
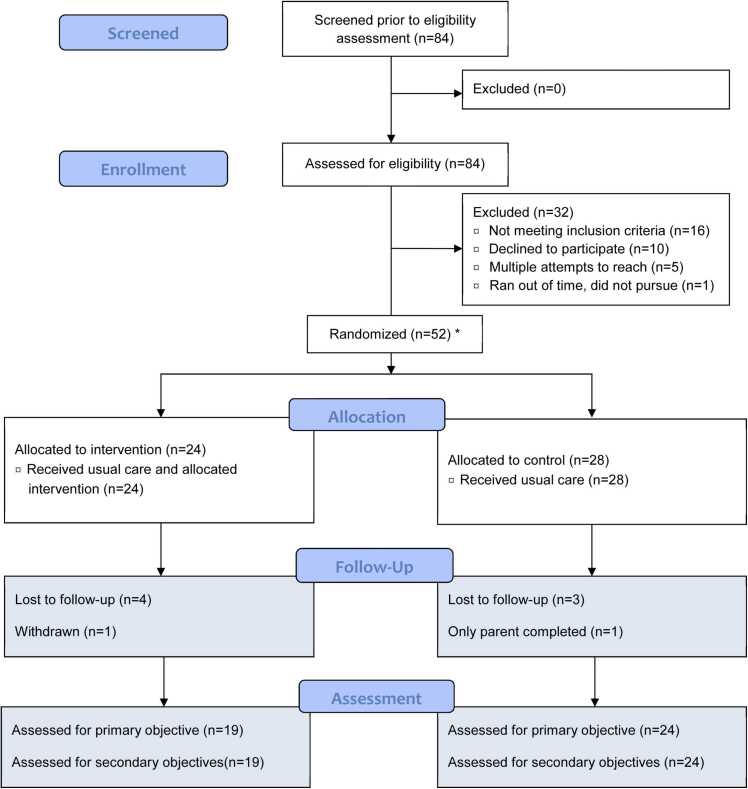
CONSORT extension for Pilot and Feasibility Trials Diagram: Study screening, enrolment, allocation, retention, and assessment.

The Consolidated Standards of Reporting Trials (CONSORT) extension to randomized pilot and feasibility trials^22^ diagram in Fig. 2 summarizes RCT screening, enrolment, allocation, retention, and assessment. Overall, 84 participants were assessed for eligibility, of whom 52 (62%) were clinic-referred and 32 (38%) were self-referred after learning about the study online. Forty-three participants were randomized and completed baseline and 6-month follow-up assessments (mean age 15.9 ± 0.8 years; 19 (44%) intervention, 24 (56%) control). Similarly, the distribution of gender was comparable between the groups (54% girls/young women). Participants in all five diagnostic groups were included, with autism spectrum disorder, cerebral palsy, and epilepsy being the most common diagnoses. Youth with multiple diagnoses were prompted to choose the one they considered as the primary diagnosis. Further demographic information is summarized in Table 2, as are baseline responses to all outcome measurements in both the intervention and control groups. Parent/caregiver participants were most commonly women (88%), and the groups were comparable regarding parental educational level.

**Table 2:** Summary of RCT patient demographics and outcome measures at baseline.

Variable	Overall (all data) (n = 52)		Overall (analysed data) (n = 43)	Group	
				Intervention (n = 19)	Usual Care Control (n = 24)
***Youth Demographics***					
**Age (years); mean (SD)**	16.0 (0.8)	15.9 (0.8)		16.0 (0.8)	15.9 (0.9)
**Gender; n (%)**					
Girl/Young woman	27 (51.9)	23 (53.5)		10 (52.6)	13 (54.2)
Boy/Young man	21 (40.4)	16 (37.2)		7 (36.8)	9 (37.5)
Other	4 (7.7)	4 (9.3)		2 (10.5)	2 (8.3)
**Diagnosis; n (%)**					
Autism spectrum disorder	16 (30.8)	15 (34.9)		8 (42.1)	7 (29.2)
Cerebral palsy	15 (28.8)	11 (25.6)		4 (21.0)	7 (29.2)
Epilepsy	13 (25.0)	11 (25.6)		5 (26.3)	6 (25.0)
Fetal alcohol spectrum disorder	5 (9.6)	4 (9.3)		1 (5.3)	3 (12.5)
Spina bifida	3 (5.8)	2 (4.7)		1 (5.3)	1 (4.2)
**Highest level of education completed; n (%)**					
High school graduate	3 (5.8)	2 (4.7)		1 (5.3)	1 (4.2)
12th grade, no diploma	3 (5.8)	3 (7.0)		1 (5.3)	2 (8.3)
10th - 11th grade	28 (53.9)	23 (53.5)		13 (68.4)	10 (41.7)
8th - 9th grade	17 (32.7)	15 (34.9)		4 (21.0)	11 (45.8)
**Health literacy; mean (SD)**					
NVS	3.5 (1.9)	3.5 (1.8)		4.0 (1.5)	3.1 (2.0)
**Region; n (%)**					
Alberta	10 (19.2)	8 (18.6)		3 (15.8)	5 (20.8)
Ontario	29 (55.8)	25 (58.1)		13 (68.4)	12 (50.0)
Quebec	6 (11.5)	4 (9.3)		1 (5.3)	3 (12.5)
Maritimes	7 (13.5)	6 (14.0)		2 (10.5)	4 (16.7)
***Parent Demographics***					
**Gender; n (%)**					
Woman	44 (84.6)	38 (88.4)		19 (100.0)	19 (79.2)
Man	4 (7.7)	2 (4.7)		0 (0.0)	2 (8.3)
**Marital status; n (%)**					
Never married	3 (5.8)	3 (7.0)		1 (5.3)	2 (8.3)
Married	36 (69.2)	30 (69.8)		16 (84.2)	14 (58.3)
Common law	4 (7.7)	4 (9.3)		2 (10.5)	2 (8.3)
Divorced/separated	5 (9.6)	3 (7.0)		0 (0.0)	3 (12.5)
**Ancestry/Ethnicity; n (%)**					
European	42 (80.8)	35 (81.4)		19 (100.0)	16 (66.7)
East and South Asian	2 (3.8)	2 (4.7)		0 (0.0)	2 (8.3)
North American Aboriginal	2 (3.8)	1 (2.3)		0 (0.0)	1 (4.2)
Latin Central and South American	1 (1.9)				
Other	3 (5.8)	3 (7.0)		0 (0.0)	3 (12.5)
**Highest level of education completed; n (%)**					
High school graduate or GED	3 (5.8)	2 (4.7)		1 (5.3)	1 (4.2)
Some college credit but no degree	6 (11.5)	6 (14.0)		3 (15.8)	3 (12.5)
Occupational/technical/ vocational program	9 (17.3)	7 (16.3)		2 (10.5)	5 (20.8)
Associate degree	1 (1.9)	1 (2.3)		0 (0.0)	1 (4.2)
Bachelors degree	22 (42.3)	16 (37.2)		8 (42.1)	8 (33.3)
Masters degree	5 (9.6)	5 (11.6)		3 (15.8)	2 (8.3)
Doctoral degree	2 (3.8)	2 (4.7)		1 (5.3)	1 (4.2)
Not specified or prefer not to say	2 (3.8)	2 (4.7)		1 (5.3)	1 (4.2)
***Household demographics***					
**Gross family income; n (%)**					
Under $15,000	2 (3.8)	1 (2.3)		0 (0.0)	1 (4.2)
$35,000 to $49,999	3 (5.8)	3 (7.0)		2 (10.5)	1 (4.2)
$50,000 to $74,999	3 (5.8)	3 (7.0)		0 (0.0)	3 (12.5)
$75,000 to $99,999	9 (17.3)	5 (11.6)		4 (21.0)	1 (4.2)
$100,000 to $199,999	18 (34.6)	17 (39.5)		9 (47.4)	8 (33.3)
$200,000 and over	4 (7.7)	3 (7.0)		1 (5.3)	2 (8.3)
Not specified or prefer not to say	11 (21.2)	9 (20.9)		3 (15.8)	6 (25.0)
**Number of dependents; median (min, max)**	2 (1,7)	2 (1,7)		2 (1, 5)	2 (1, 7)
**Household dynamics; n (%)**					
Both parents	32 (61.5)	26 (60.5)		14 (73.7)	12 (50.0)
Parent and stepparent	3 (5.8)	3 (7.0)		2 (10.5)	1 (4.2)
Single parent	9 (17.3)	7 (16.3)		1 (5.3)	6 (25.0)
Adoptive parent(s)	4 (7.7)	4 (9.3)		2 (10.5)	2 (8.3)
**Custody; n (%)**					
Full custody	27 (51.9)	23 (53.5)		11 (57.9)	12 (50.0)
Shared/joint custody	8 (15.3)	6 (14.0)		2 (10.5)	4 (16.7)
***Outcomes***					
**TRAQ self-management; mean (SD)**	2.2 (0.5)	2.2 (0.5)		2.3 (0.6)	2.2 (0.5)
Missing	1	1			1
**TRAQ self-advocacy; mean (SD)**	3.1 (0.6)	3.2 (0.6)		3.4 (0.6)	3.0 (0.5)
Missing	3	1			1
**PedsQL**^**TM**^**(Overall); mean (SD)**	65.1 (18.2)	64.6 (18.8)		64.5 (16.5)	64.6 (20.8)
Physical health summary	65.8 (27.4)	65.9 (27.9)		69.4 (22.2)	63.2 (31.9)
Psychosocial health summary	64.8 (18.6)	64.0 (19.2)		61.8 (21.6)	65.6 (17.3)
Emotional functioning subscale	61.8 (22.3)	60.1 (22.4)		54.2 (24.3)	65.0 (19.9)
Missing	1	1			1
Social functioning subscale	68.2 (22.2)	67.5 (23.3)		68.4 (24.4)	66.7 (22.9)
School functioning subscale	62.8 (20.7)	62.9 (21.7)		62.1 (24.2)	63.6 (20.0)
**TRANSITION-Q; mean (SD)**	56.6 (10.2)	56.5 (10.2)		57.0 (10.1)	56.1 (10.4)
**COPM-P; mean (SD)**	4.9 (2.0)	4.9 (2.1)		4.3 (1.8)	5.3 (2.2)
**COPM-S; mean (SD)**	5.0 (2.1)	5.0 (2.1)		4.8 (2.1)	5.2 (2.2)
**NVS; mean (SD)**	3.5 (1.9)	3.5 (1.8)		4.0 (1.5)	3.1 (2.0)
**MPOC scores; mean (SD)**					
Enabling and partnership	4.4 (1.5)	4.4 (1.5)		4.6 (1.5)	4.3 (1.6)
Missing	3	3		1	2
Providing general information	3.6 (1.7)	3.5 (1.6)		3.4 (1.6)	3.6 (1.7)
Missing	5	5		3	2
Providing specific information	4.4 (1.5)	4.3 (1.5)		4.2 (1.5)	4.3 (1.4)
Missing	3	3		1	2
Coordination and comprehensive care	4.8 (1.3)	4.8 (1.4)		4.8 (1.5)	4.7 (1.3)
Missing	3	3		1	2
Respectful and supportive care	5.1 (1.1)	5.1 (1.1)		5.1 (1.1)	5.1 (1.1)
Missing	3	3		1	2

SD=Standard deviation; TRAQ=Transition Readiness assessment Questionnaire; PedsQL™ =Pediatric Quality of life; COPM-P = Canadian Occupational Performance Measure-Performance; COPM-S=Canadian Occupational Performance Measure-Satisfaction; NVS= Newest Vital Sign; MPOC=Measure of Process of Care.

As part of the embedded qualitative study, seven youth (3 boys/young men, 3 girls/young women, 1 non-binary) and five parents (all mothers) consented and were interviewed. Table 3 summarizes demographic information for the families who participated in the qualitative interviews. The participants who agreed were from Ontario (5 youth, 3 parents) and Alberta (2 youth, 2 parents). There was one parent whose child declined to be interviewed; however, all other qualitative participants were parent-child dyads.

**Table 3:** Youth demographics for qualitative interview participants.

	Respondent			Diagnosis	Region	Age	Gender	TRANSITION-Q Score (BL)
	Parent	Youth						
1	x		x	ASD	Ontario	16	M	44
2			x	ASD	Ontario	16	Non-binary	70
3			x	CP	Ontario	16	F	58
4	x		x	ASD	Ontario	17	M	70
5			x	ASD	Ontario	15	F	41
6	x		x	FASD	Alberta	17	M	47
7	x		x	ASD	Alberta	15	F	56
8	x			ASD	Ontario	15	M	44

### 3.2 Process, resource, and management feasibility outcomes

In Table 1, we summarize key feasibility and App engagement outcomes, criteria for success, method of analysis and results. None of the regions recruited at the planned minimum rate of four participants per month (Table 1). Across recruitment sites, the time from initial ethics application to approval notification was over six months (median = 210 days, Table 1) with extraordinary delays greater than 15 months in two sites. The criterion for greater than 90% uptake of the App was met with 23/24 = 96% (0.8, 1.0) downloading the App on their device and completing orientation (Table 1). As illustrated in Table 1, we did not meet our retention criterion for greater than 90% of participants: 43 of the 52 randomized participants (83% (0.7, 0.9)) completed the 6-month follow-up visit.

### 3.3 Youth experience and satisfaction with the App, and opportunities for improvement

Table 4 summarizes the qualitative data collected from youth and parents/caregivers to facilitate exploration of the use (technical glitches, reminders and cues), perceived value (relevant to transition, applicable to me), experience (navigation, look and feel, integration), and satisfaction (customization) with the App and to inform ongoing App development. Key qualitative findings suggest that to improve youth engagement with the App, more customization and choices should be offered, allowing non-linear navigation and the ability to select relevant content based on current needs. While most youth appreciated the ease of navigation and interactive cues, some felt the design was childlike and overly educational, impacting their interest. Interview data also indicated a role for parents and healthcare providers in encouraging use and helping youth connect App content with real-world applications. Additionally, the App’s functionality could be enhanced for mobile use, addressing technical issues like glitches and video freezing for a more seamless experience.

**Table 4:** App use, perceived value, experience, and satisfaction results from qualitative interviews with youth (Y) and parents (P).

Construct	Explanation	Quote
**App Use**		
Technical glitches	The App could be better optimized for mobile use. Only one youth in the study was not able to get the App working on their device at all. Some had problems with videos freezing or the App glitching or crashing. Some reported that closing it and opening it again resolved the issue. Some reached out for support and others never re-gained access.	*“Occasionally I would get something like a glitch where I couldn't play a video and I'd have to close and restart it again.” Y2* *“It would kind of just stall there…Yeah, it's too bad, but it just didn't work so well on his phone. I think he's a kid who would've, he would've enjoyed using it.” P4*
Reminders	Continuous reminders from multiple sources (within the App, and from parents, etc.) could help youth establish a routine and continue using the App.	*“I'm terrible at keeping a routine, but also at school I had to get caught up on a lot of work and then I like didn't have time to do it. And then I stopped doing the routine and then I did it less and less.” Y2* *“He has a difficult time like remembering to do things… I don't think there was anything sort of set up to for him to cue him to do it…even if it could be set up in a way that like the parent could be connected so that I would get a reminder to then remind him or that we both got a reminder, you know, something like that I think would be helpful.” P1*
Cues	Parents can help cue youth to think about how parts of the App may be useful to the things they need to do next. Parents were not very involved in using the App with their child in this study. Some helped with setting it up or troubleshooting technical glitches.	*"He needs, it needs to be continuous like, oh, well it would be useful for me to go back and look at, oh now I'm meeting, like now I'm going to my family doctor. So, what do I need to know about that?" P1*
**App Perceived Value**		
Relevant to transition	Overall, youth and parents who reviewed the App told us the information provided was comprehensive, relevant and useful to prepare youth for health care transition.	*“Even just the little bit that I did do did kind of give me some information that I needed in order to be able to do some of that stuff on my own.” Y5* *“Like preparing the right way, like, you know, like knowing about yourself to be able to answer like the doctor's questions and stuff like that, yeah that's like good. And I don't know, like just, I don't know, like just all of it would be like super like helpful.” Y3* *“I think the information is necessary for him because he is shortly transitioning into adult care” P1*
Applicable to me	When asked about what they needed to get ready for health care transition, youth were often not able to see a connection between what they needed and what the App had to offer. However useful or relevant, youth may not engage if they perceive the information as not currently applicable or relevant for them. Parents [and healthcare providers] through collaborative discussions may be able to help coach and work with youth to better incorporate the App into care and to help apply the skills in real world situations.	*“I don't struggle with some of the same things that other people do. So, like certain aspects of it, I'm like, okay, like this doesn't apply to me. Like I know how to take care of myself…So, it's not really as like relevant to me.” Y* *“In terms of transition, I’m kind of self-taught…I had a lot of resources…but for me currently, based on my level of function, I don’t think it was meant for me.” Y* *“That's also like important information, especially things like medications because chances are, even if they don't take medications at some point, they're going to need an antibiotic, or something is going to come up and they might need to start taking medication. So even if they don't regularly… but I mean I know teenagers don't always think that way.” P6* *“When you get someone who's speaking [in the videos] who has your diagnosis, it's like, yay. I don't know. I just felt kind of happy, but I think it's just easier to process what they're saying when like someone's actually speaking and it's not just text”. Y2* *"He just has a hard time with like initiation and transferring that information from the app into kind of a real-life situation." P1*
**App Experience**		
Navigation	Most said it was easy to move through the various features and tools in the App, and that they liked the cues in the App to help them make their way through the visits (e.g., flashing items to tell them where to click next).	*“It was pretty like easy to navigate, like it made sense. The only thing I think that was a little bit challenging is that my phone was so small that like, when you had to like scroll to move around the city, that kind of got a little bit disorienting a little bit to me …but like, other than that, it was like, it's pretty like straightforward…I think honestly it was a really good balance of just like everything. Like you weren't just like watching videos whole time. You weren't just like reading things the whole time… it was a nice balance of like different activities, but yet you kind of like learned this, like whatever, like you learned at the same time as doing all this other stuff.” Y3*
Look and feel	Some felt the look and feel was childlike, the graphics were low level and educational. If App content is too educational, or too much like school and not fun, youth may not use it.	*"I felt like it was kind of childlike a little bit, but I understand that it's supposed to help people transition, you know, from a child hospital to an adult hospital. So, it's obviously supposed to be for young people as well. But I do feel like if you're on the older scale of the people using it, it does kind of feel like, oh, I don't know." Y2* *“I don't think it was like, you know, like top of the line…honestly, it's probably just like the nature of what you're trying…like, it's supposed to be like educational and it's supposed to have that certain feel” Y3* *“I would say it was the graphics were kind of grainy and, it was just kind of pixelated and it was, you know, it just seemed underdeveloped…there's the town…the layout of it and the different places where you would go to learn was kind of appealing to you and, or something that you found useful about the app.” Y4* *"If it looks like school and he's not in school, it's not fun." P8*
Integration	Suggestion that incorporating digital, interactive forms that could be used in health care visits would be of value.	*“When you're talking about transition, you're also talking about sort of how to navigate the medical system…maybe making it easier somehow through the app to fill out forms and pages, like some sort of digital representation…having a virtual form in the app that represents a real form that would be easier for someone with a disability, a disabled person to sign and to manage.” Y4*
**App Satisfaction**		
Customization	To improve App satisfaction and experience for youth, more choice and customization is recommended, offering a balance between required content and information youth can choose based on their current questions and needs. Preference to skip or choose the order and timing of the content (non-linear).	*“Transitioning, really depends on the individual a lot of the time. It depends on the, you know, life experience and their abilities and whatnot, and their social and geographical environment. So, I think you have to sort of customize it to the individual” Y4* *“Some that's like, you know, I'm not like mandatory but you know, like automatic that you go through and then others that it's more optional. Like if this applies to you, if not, skip. I don't know.” P6* ***“If you don't have to kind of go through it in a linear way if you can just kind of pop into the area that you is important to you…I think that would be much better**” P1* *“At least for my son, and I don't think he is unique in this way, instant gratification and instant information is where he lies. So, if he could cherry pick the information that was important to him, let's say he was great at something else and having to do the work to get to the next part was just like, ugh, like not interested. I just want to know this. Why can't I get to that part? … if there was a catalog…a way to select different sections and it was categorized and maybe in each category you, there was like some sort of step by step where you had to complete each level, if you will, within the category, that would be, I think that would probably be successful.” P8*

### 3.4 Research staff experience of study feasibility and impact of COVID−19 pandemic

The focus group with RA staff (Supplemental File 6) and the electronic survey of HCPs (Supplemental File 7) revealed information about their experiences related to feasibility (the internal processes, resources, and management of the study), and about the impact of the COVID−19 pandemic. Relevant findings are synthesized in Table 5, together with lessons learned and practical implications.

**Table 5:** Synthesizing Feasibility Lessons: Recruitment, App Use, Clinical Workflow Integration, and Engagement.

Feasibility aspect	Lessons from Research Assistants (RAs) [Supplemental File 6]	Lessons from Health-care Providers (HCPs) [Supplemental File 7]	Lessons from Youth & Parents – (Table 4)	Practical implications
**Recruitment channels & context**	Recruitment was the most challenging due in part to COVID−19; in-person conversations were more successful than virtual; multiple strategies (clinics, community, social media) and advance ethics approval for diverse channels would help. Parental concerns about extra screen time reduced interest.	Virtual care and lack of an on-site RA reduced recruitment opportunities; texting/emailing youth could help; parents’ involvement can aid recruitment.	Youth reported variable relevance and motivation to participate; reminders/cues would help sustain engagement once recruited.	For future trials: plan ahead for ethics approval to use mixed recruitment strategies (clinic + digital) and pair outreach with automated reminders and parent-supported prompts.
**Screening & eligibility**	TRANSITION-Q cutoff sometimes felt out of range for younger youth; disappointment when just below cutoff; re-testing could bias responses.	--	Youth vary widely in skills; some perceive content as not applicable if already self-managing.	Consider broader inclusion or tiered tracks (by readiness/needs) to avoid excluding those who could still benefit from targeted modules.
**Retention & follow-up**	Retention was respectable but varied with timing; better incentives and ongoing communication (multi-modal: phone/email/text) improved completion of 6-month questionnaires.	--	Youth/parents emphasized need for reminders and shared prompts (youth + parent) to keep a routine.	Build automated in-app reminders with optional parent co-notifications to support retention.
**Adherence to the App**	RAs were removed from day-to-day App use; planned encouragement emails didn’t go out consistently due to CMS tracking access; multi-device access and push messages could help.	--	Youth faced mobile glitches; some lost access and didn’t re-engage; reminders would help.	Implement push notifications, reliable dashboard tracking, and cross-device logins; ensure tech reliability on phones.
**App Technical performance**	Tech glitches frustrated some youth (especially those with attention challenges), jeopardizing re-engagement; offering backup written resources was suggested.	--	Reports of videos freezing/crashing; one youth couldn’t get the app to work; closing/reopening sometimes fixed it.	Prioritize mobile optimization, better error recovery, and offline/backup access to content.
**Integration into clinical workflow**	Online options made data collection easier; RAs found sharing screens and meeting youth “where they are” online helpful.	HCPs see the App as a valuable teaching tool for a subset (particularly youth who are higher functioning); propose use in waiting rooms or pre-visit; use Transition-Q to start goal-oriented conversations; some perceived greater youth confidence in appointments.	Youth suggested adding digital forms linked to real clinical paperwork; navigation was generally easy.	Embed App into visits: pre-visit assignments, waiting-room use, and goal-setting during clinical encounters; consider in-app form completion to bridge clinic/admin tasks.
**Motivation & engagement**	Technical issues and early frustration reduced willingness to try again; encouragement emails were intended but inconsistent.	Youth prefer interactive/gamified elements; reading-heavy curriculum was less appealing.	Parents/youth want customizable, non-linear paths and the ability to “cherry pick” topics; look/feel sometimes felt childlike.	Increase interactivity, add non-linear, customized navigation, and tailor aesthetics to older teens/young adults; ensure early wins to maintain momentum.
**Parent role**	RAs often began with both youth and parent present for recruitment; proximity helped clarify details; newsletters were sent once to families.	HCPs note parent engagement can support recruitment and integration (dual participation suggested).	Parents can cue youth to apply content to real tasks (e.g., “meeting family doctor”), desire for linked reminders to both youth and parent.	Offer an optional parent portal/coach mode with shared reminders and visit prep cues.
**Resources & staffing**	Flexible scheduling (evenings/weekends) was often required; on-site presence boosted recruitment; social-media promotions had modest yield.	Lack of on-site RA hampered recruitment; virtual care shifted provider roles, reducing direct recruitment touchpoints.	--	Budget for on-site recruitment bursts around clinic days; complement with targeted, not just broad, digital outreach.
**Ethics & admin timelines**	Multi-site REB/contract delays compressed recruitment timeline; streamlined provincial processes help but can still bottleneck; bilingual (French) content was a requirement in Quebec Region.	--	--	Start ethics/contracts early and seek approvals for multiple recruitment methods from the outset; plan timelines for translations.
**Data collection operations**	REDCap worked well; auto-reminders helpful; screen-sharing to read items together built rapport; minor glitches were fixed.	--	--	Maintain centralized data tools, scripted rapport-building, and operations checklists for consistency across sites.

### 3.5 App engagement outcomes

**Fig. 3:**
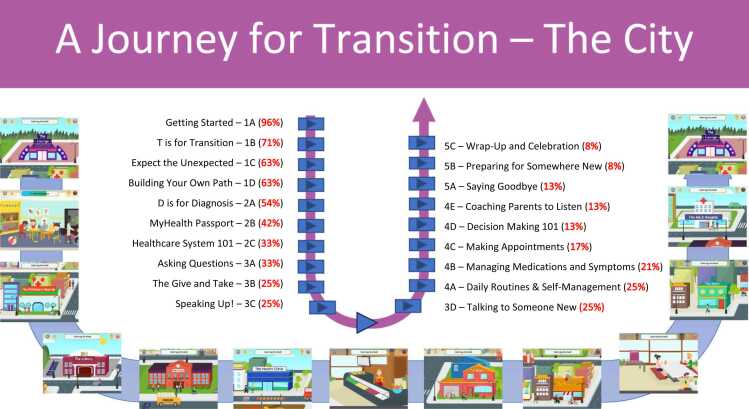
Percentage of participants in the intervention group who completed each visit in the App (n = 24).

We observed modest engagement with the App despite 96% uptake; intervention participants logged into the App an average of 11.2 times ± 9.5 (Table 1). Fig. 3 shows the percentage of participants who completed each session in the App, with fewer than half progressing further than the fifth session out of 19 total in the App. Five (21%) participants contacted technical support; seven (29%) used the App on an Android phone and 17 (71%) used it on an iPhone, iPad, or MacBook. With a mean score of 65.4 ± 26.8, we did not meet the criterion for acceptable usability or experience of the App (defined by a mean system usability scale score greater than 70);^23^ for example, youth ability and confidence to use the App, how easy it was to use and whether all the parts of the App worked well together (Table 1).

### 3.6 Scientific feasibility outcomes: Exploring estimated treatment effects

No adverse events were reported during the pilot RCT. No serious illnesses were observed at 6 months post intervention in either the intervention or usual care control groups as measured by hospitalizations, doctor visits, and long-term care admissions. In terms of change in transition readiness, in both the intervention and usual care control groups, there was an improvement in TRAQ self-management score at 6 months post intervention, from 2.3 ± 0.6 (standard deviation SD) to 2.5 ± 0.7 (SD) and 2.2 ± 0.5 (SD) to 2.5 ± 0.9 (SD), respectively (Supplemental File 8). The estimated treatment effects, expressed as adjusted mean differences between the intervention and control groups at 6 months, were small and not statistically significant. For TRAQ self-management, the intervention group scored on average 0.16 points lower than the control group (mean difference −0.16, 95% CI: −0.56–0.24). For TRAQ self-advocacy, the difference was essentially zero (mean difference 0.003, 95% CI: −0.35–0.36). These estimates were derived using ANCOVA models adjusted for baseline values and region. The confidence intervals for both outcomes include zero, indicating no clear evidence of a treatment effect.

Initial treatment effects on secondary outcomes of health and use of health systems were also assessed and are included in Supplemental File 8. Participants set life-course goals at baseline according to the three COPM categories of self-care, leisure, and productivity (Supplemental File 9). In the intervention group, productivity goals were identified most frequently (32/80, 40%) followed by self-care (28/80, 35%) and leisure (20/80, 25%) goals. Within the self-care category, only 5 (26%) in the intervention group and 12 (50%) in the control group prioritized managing physical and mental health. There was an increase in both COPM performance and satisfaction scores at 6 months post intervention for both allocation groups (Supplemental File 8).

## 4 Discussion

### 4.1 Feasibility challenges

Our full, patient-oriented RCT was designed to assess the effects of the MyREADY Transition™ BBD App based on the premise that recruitment of youth with BBD was feasible. Challenges with implementation of the protocol and limited feasibility led to the trial being stopped early (Fig. 1), at only 16% of its predetermined number of participants. In addition, we observed challenges with App engagement. Similar interventions have been attempted in chronic illness with similar challenges.^24^ Our RCT internal pilot evaluation captured quantitative and qualitative data on key factors, informing the development of HIT interventions and the design of healthcare transition studies targeting youth with chronic health conditions, including those with BBD.

The a-priori criteria for process feasibility (recruitment of 4 participants per month per region) was not met with an observed mean rate of only one-fourth of what was planned, varying between 0.7 (Maritimes) to 1.5 (Ontario). Significant delays in the recruitment rates were curtailed by the COVID−19 pandemic. The shortfall prompted adaptations to the original protocol,^1^ making concurrent adaptations to increase the recruitment success, including community and online recruitment strategies championed by our PFAC partners, which ultimately accounted for 35% of the recruited sample. Challenges in the recruitment of adolescents with chronic conditions, including those with BBD, for healthcare transition studies is well documented.^25^, ^26^, ^27^ Contributing factors include age, disease complexity, limited altruistic motivations, prior recruitment to other studies and research fatigue.^27^ Given these ongoing recruitment challenges, further study is needed to build on lessons learned in studies like ours, such as seeking ethics approval from the outset for multiple recruitment strategies; investing in direct personal connections and building these connections over time; and setting deadlines (Supplemental File 6).

Challenges with management feasibility were reflected in the time to ethics approval after proposal submission. While acknowledging there were institutional priorities for research ethics boards during the time of this study (i.e., focus on COVID−19 research applications and the subsequent backlog), further work is needed to create greater efficiencies provincially and nationally to streamline the ethical approval process for multi-institutional research trials.

Resource feasibility (as measured by participant retention and App uptake) was modest. Youth randomized to the intervention arm who were successful in downloading the App and completing the first session in the App completed less than a third of the App visits (Fig. 3). Similar to our findings, a distinct feature of e-health trials is the substantial number of participants who drop out or stop using the intervention. Eysenbach^28^ notes that discontinuation of e-health interventions in trial settings is common and warrants reporting, as closer analysis of attrition data may provide insights for maximising adoption in "real-world" settings.

### 4.2 App Engagement

Quantitative data on usability and experience of the App was modest. However, together the quantitative and qualitative evaluation of the App use in this study, yielded valuable recommendations for alternative ways to organize or package the App content, which may improve user engagement with the App. For example, the curriculum could allow a more self-directed approach where youth choose learning topics in any order, such that they can focus on topics relevant to them in the moment. Although there is a trade-off with requiring external support/resources versus a standalone patient-facing intervention, another recommendation was to better integrate the App into care by involving parents and HCPs as coaches working in collaboration with youth. This notion is supported by previous literature suggesting that parents want to be involved in the transition process^29^ and that parents and HCPs should partner in enhancing capacity and building skills to support youth through transition^30^ A key lesson learned is that acquiring transition knowledge, even with virtual "practice" does not and cannot replace human support.

### 4.3 Aligning interventions with youth developmental readiness for change

To inform future studies, we report on the baseline and follow-up data, along with the initial treatment effects of our patient-facing App. We observed that the TRAQ detected change over time. Sawicki et al.^14^ describe the stages of change model for TRAQ development, mapping scores to developmental stages of readiness to transition, which is useful in choosing appropriate interventions based on the skills assessed. Importantly, the baseline TRAQ self-management score of 2.2 ± 0.5 overall amongst participants in this study maps onto a response category of “I do not know how but I want to learn” or the contemplation stage of change “intends to take action in the next 6 months”. This does support the use of our eligibility screen for baseline threshold of readiness on the TRANSITION-Q as an appropriate measure to identify participants who were developmentally ready to benefit from the App’s content. It is possible that with adequate power, an improvement in TRAQ score may have been observed over time.

### 4.4 Limitations

Limitations should be considered when interpreting the findings. We did not control for RA attention between the groups, with youth in the intervention group having more interaction with the RA. It is difficult to determine how representative our sample is with multiple recruitment methods (clinic, online, community). The results may be affected by bias, particularly self-selection at study entry. Also, the qualitative findings are limited to only 8 youth, 6 of whom have autism spectrum disorder, hindering generalizability to other disabilities. Moreover, the threshold for transition readiness excluded those who showed full dependence on caregivers.

### 4.5 Conclusions

Despite the MyREADY Transition™ BBD App being carefully developed using an Agile Iterative methodology, in partnership with patients and families, and informed by cognitive learning theory, we observed only modest App engagement among youth overall. When interviewed about what they needed to prepare for transition, youth often identified some of the App content, but importantly, were not able to make the connection to how the App could help them develop the required knowledge and skills to prepare for healthcare transition. As the preparation for healthcare transition in adolescents includes both acquiring knowledge and developing self-management skills, future research should consider youth motivation (throughout the continuum of contemplation, preparation, and action stages of change) as a crucial factor when planning and implementing transition strategies.^31^

While we did not proceed to the full-scale RCT, this pilot has provided critical insights into the process, resource, management, and scientific feasibility, as well as App engagement of the MyREADY Transition™ BBD App designed to enhance healthcare transition readiness in youth with BBD. The lessons learned can inform further development of this and other digital tools, as well as the design and implementation of future pragmatic evaluation studies, ensuring that they are both methodologically robust and more likely to achieve the desired healthcare transition outcomes. As generative artificial intelligence (GAI) advances, it may play a role in future development of digital tools. Future research will continue to optimize the App to better meet the needs of youth transitioning to adult healthcare, for BBD and for populations with other chronic health conditions.

**The trial is registered with clinicaltrials.gov (NCT03852550)**

## Declaration of Generative AI and AI-assisted technologies in the writing process

During the preparation of this work the authors used Microsoft 365 Copilot in order to help condense the abstract to 250 words, to draft Fig. 1 (design pivot) and Table 5 (synthesizing feasibility lessons). After using this tool, the authors reviewed and edited the content as needed and take full responsibility for the content of the published article.

## Ethical Statement

The study has been approved by the Research Ethics Board of each participating site: Hamilton Integrated Research Ethics Board (Clinical Trials Ontario #1666), (Alberta) Health Research Ethics Board (Health Panel #MS2_Pro00086027), Horizon Health Network Research Ethics Board (#2018–2689), Research Ethics Board at the University of New Brunswick (Saint John #037–2019), Mount Allison University Research Ethics Board (#102606) and IWK Research Ethics Board (#1 025 247).

## Funding sources

CHILD-BRIGHT Network funding (CIHR-SCA−145104). Funding partner support from Montreal Children’s Hospital Foundation, Faculty of Health Sciences of McMaster University, 10.13039/100013123New Brunswick Health Research Foundation, McMaster Children’s Hospital Foundation, 10.13039/100008360Hamilton Health Sciences, and Centre hospitalier universitaire mère-enfant Sainte-Justine.

The funder of the study had no role in study design, data collection, data analysis, data interpretation, or writing of the report.

## Declaration of Competing Interest

AM and JWG received research grants from the Canadian Institutes of Health Research Strategy for Patient-Oriented Research. AM holds grants from the Heart and Stroke Foundation of Canada and the Fonds de recherche du Québec–Santé, where she is a senior clinical research scholar. JWG held the Scotiabank Chair in Child Health Research (2013–2021). BG, LN, SS, and AV-DL were paid for their work as project staff members. The remaining authors declare that the research was conducted in the absence of any commercial or financial relationships that could be construed as a potential conflict of interest. No funders had a role in the design and conduct of the study.

## Data Availability

Data will be made available on request.
