# Supplementary material for: An e-health transition intervention for youth with brain-based disabilities: Pilot and feasibility results from a Randomized Controlled Trial
Source: Health Care Transit. 2026 Jun 10;4:100144. doi: 10.1016/j.hctj.2026.100144 (PMC13273774; doi:10.1016/j.hctj.2026.100144)
Supplement: Supplementary material [file mmc1.pdf]

Supplemental File 1. Youth Reference Handout, Intervention Group.

READYorNot™
Brain-Based Disabilities Trial

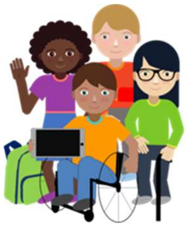

Reference Handout for Youth

The MyREADY Transition™ BBD App is for youth...

- who are 15, 16 or 17 years old.
- with autism spectrum disorder, cerebral palsy, epilepsy, fetal alcohol spectrum disorder or spina bifida.

The App was created to help youth start to get ready for health care transition. Health care transition is when you make the change from getting pediatric services (e.g., from the children's health care team, the children's hospital or the children's treatment centre) to looking after your own health in adult services.

In this study we really want to see how youth use the App by themselves. You can always get help from our support website at www.myreadytransitionbbd.app. And, it's okay to ask your parent/caregiver if you are stuck or if you need help answering a question in the App.

Navigating the App

There are 19 parts in the App. We suggest that you make your own routine for using it. The Trophy Case will show the completed parts.

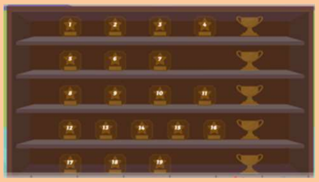

Example:

- 1 part each day → 19 days to do all of the App.
- 1 part each week → 19 weeks to do all of the App.

1

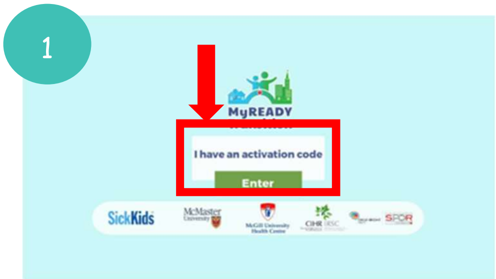

Log in with the activation code

2

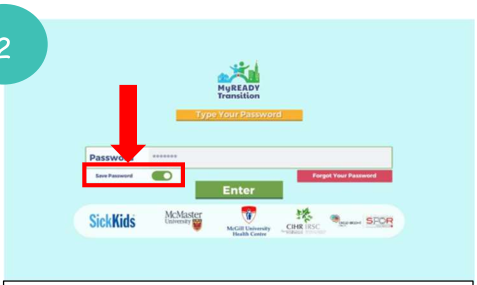

Click to save the password

3

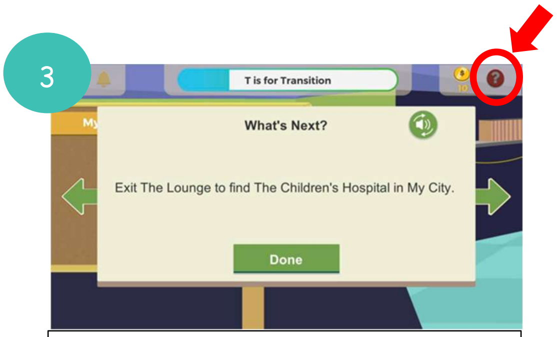

If you get lost, click on the "What's Next?" button

4

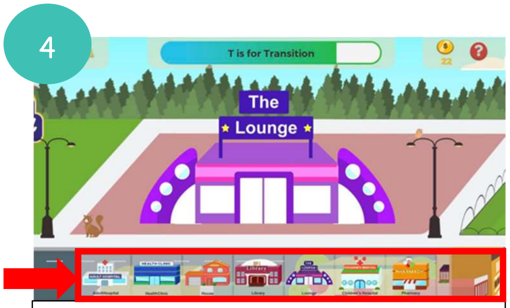

Click on the icons to go to different buildings

Remember to connect to the Internet to use the following features:

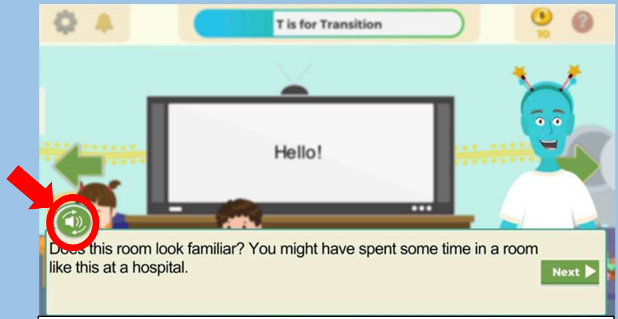

Click on the icon to hear the text

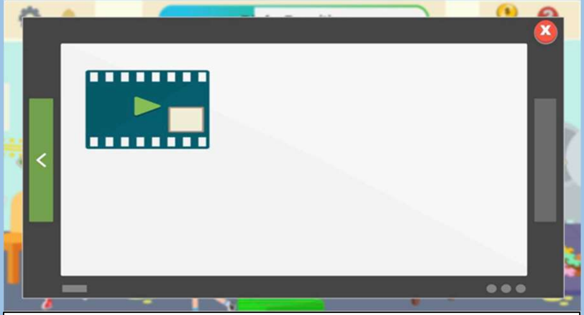

Watch videos

Bulletin Board

Provides resources and tools

Click on the arrows to move through the different resources

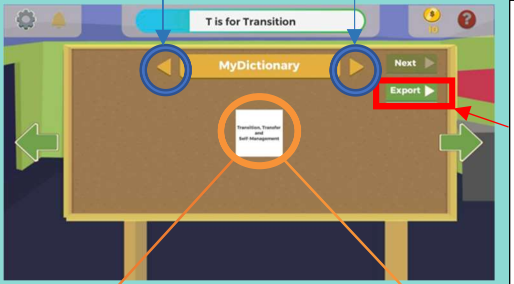

Click "export" to open up your email and send the resource to yourself

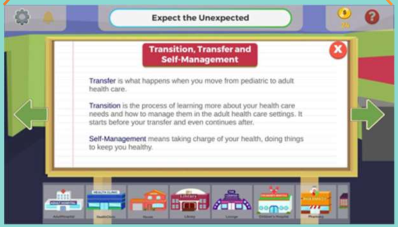

Click on the resource to review information

Whiteboard

Summarizes information

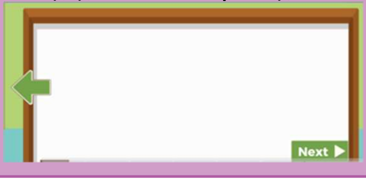

Game Room

Play games with the coins you earn

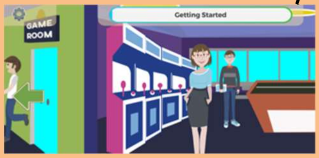

Countdown Timer

Lets you know when the next part is open (at least one day between the parts)

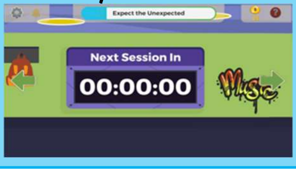

My Activation Code: ______________________________

Technical Support Information

You can always get help from our support website at www.myreadytransitionbbd.app
