# Supplementary material for: An e-health transition intervention for youth with brain-based disabilities: Pilot and feasibility results from a Randomized Controlled Trial
Source: Health Care Transit. 2026 Jun 10;4:100144. doi: 10.1016/j.hctj.2026.100144 (PMC13273774; doi:10.1016/j.hctj.2026.100144)
Supplement: Supplementary material [file mmc2.pdf]

Supplemental File 2. Health Care Provider Reference Handout.

READYOrNot™

MyREADY Transition Brain-Based Disabilities Trial

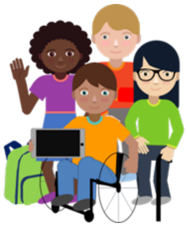

Reference Handout for Health Care Professionals

The MyREADY Transition™ BBD App is for youth...

- who are 15, 16 or 17 years old.
- with autism spectrum disorder, cerebral palsy, epilepsy, fetal alcohol spectrum disorder or spina bifida.

The App was created to help youth start to get ready for health care transition. Health care transition is when youth make the change from getting pediatric services (e.g., from the children's health care team, the children's hospital or the children's treatment centre) to looking after their own health in adult services.

There are 19 parts in the App with activities, games, and videos.

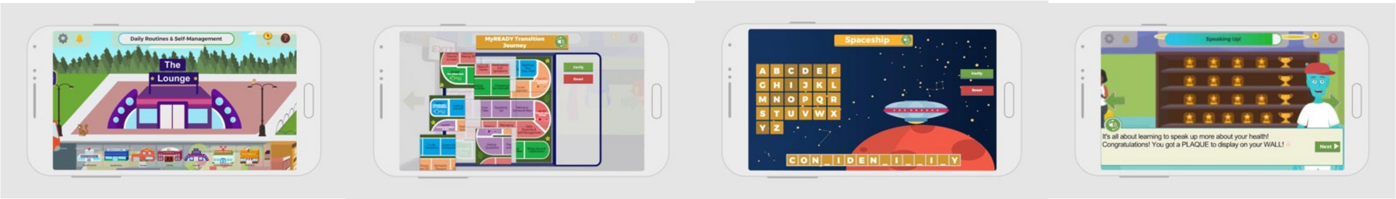

Study Purpose: We want to do this randomized control trial (RCT) to find out if there is a benefit to using the App compared to not using it. We want to see if the App will help youth with things like knowing about their health, knowing what questions to ask, and when to ask for help.

In an RCT, all youth in the study get the same care they have been getting already. Half will use the App and half will not. There's an equal chance of being in either group. After the study is done, youth in both groups will have a chance to use the App.

App support will be provided by the study team at www.myreadytransitionbbd.com. To help us understand how youth use the App on their own, we ask that health care providers are not:

- ✗ Completing any part of the App for your patients.

We understand that your patients might ask you for help while using the App. Here are some ways you may offer support for your patients in this study:

- ✓ Answering questions that they might have about their health care.
- ✓ Encouraging them to talk to you about their health.
- ✓ Discussing plans about preparing for health care transition based on topics raised in the App.
