# Supplementary material for: An e-health transition intervention for youth with brain-based disabilities: Pilot and feasibility results from a Randomized Controlled Trial
Source: Health Care Transit. 2026 Jun 10;4:100144. doi: 10.1016/j.hctj.2026.100144 (PMC13273774; doi:10.1016/j.hctj.2026.100144)
Supplement: Supplementary material [file mmc4.pdf]

Supplemental File 4. Youth Qualitative Interview Guide.

CHILD-BRIGHT READYorNot™ Brain-Based Disabilities Trial
Interview Guide

Interview Logistics

**Participant ID**	
**Interview Date**<br>(month/day/year)	
**Interviewer**	
**Length of Interview** (minutes)	
**Additional Notes**	

Introduction

Hello, my name is [name] and I am [position] on the READYorNot™ Brain-Based Disabilities Trial. Thank you for agreeing to take part in this interview. As a reminder, we are conducting this study to see if, for youth who are 15-17 years old with a brain-based disability, it is better to use the MyREADY Transition App or not to use it. We want to know if using the App can improve healthcare transition experiences (moving from the children's hospital or children's treatment centre to adult health care). You have been invited to take part in this interview because you were someone who received the App as part of the study.

The purpose of this interview is to ask you about your experiences using the App and to understand how useful you think it might be for healthcare transition. We are also interested to learn about your experience being enrolled in a study virtually. There are no right or wrong answers. The interview today will last about 30-45 minutes and will be audio-taped if you are okay with that. Your participation is completely voluntary and we can stop the interview at any time. The information you share here will be kept private. Researchers involved in the READYorNot™ Brain-Based Disabilities Trial will be able to read what you tell me, but they will not see your name attached. Do you have any questions?

INTERVIEW QUESTIONS

OPENING QUESTIONS – GETTING STARTED AND APP USE

1. *What device did you use the App on? (desktop or laptop computer, tablet, phone)? Why did you choose that device? Would you have preferred to use it on a different device? Why or why not?*

2. *Did you have any problems getting the App to work on your device?*
   Probes:
   - *Did you have any problems with the link you were provided?*
   - *Did you have any problems installing the program?*
   - *Did you have any problems with the registration process?*
   - *If you had any problems, did you seek support? How? Describe for me what happened when you asked for support? Was the problem resolved?*

3. Please tell me a little bit about how you used the App?
   Probes:
   - *Did how you used the App at the beginning change at all as you continued working through it? What changed? Please describe.*
   - *If youcontinued using it, why? If they stopped using it, why? Explore barriers and facilitators to use.*
   - *If you used the app, when did you find yourself using it? Did you use it alone or with someone else present?*

QUESTIONS ABOUT APP LOOK AND FEEL

1. Tell me what you thought of the look of the App?
   Probes:
   Was it appropriate for someone like yourself? How did you feel about the city design? The illustrations? The animations?

2. Were there parts of the App that you particularly liked or didn't like? Were there parts of the app you used a lot? Parts you didn't use at all? Why or why not?

   Probe: Mentor, Videos, Challenges, Bulletin Board, White Board, Games/Challenges, Games Room, Coins/Rewards, Text-to-speech, Different voice options

3. Do you have any recommendations for changes to the App that could make it better?

QUESTIONS ABOUT APP CONTENT

1. Can you tell me what information in the app you found most useful? Less useful?

2. Is there any other information you would like to have seen included in the App?

QUESTIONS ABOUT SKILL-BUILDING

1. Can you tell me something you learned in the App? Or can you give me an example of when you did something in real-life that you learned in the App?
   Probes:
   - *Have you used any of the Mentor's tips in real-life? Have you used any of the tips from the videos? Any of the tips from the challenges or games?*

QUESTIONS ABOUT HEALTHCARE MANAGEMENT AND DECISION-MAKING

I'm going to talk to you now about managing your own healthcare. This means doing things to take care of yourself – like the everyday things you might do at home (eating well, taking medications, exercising) or things you do for your doctor's visits (like preparing a list of questions and remembering to ask them at the appointment).

1. What sorts of skills do you think you need to do to manage your own healthcare? How has the App helped you to develop these skills?

2. How has using this App influenced the way you make decisions about your healthcare (or healthcare transitions)?
   Prompts:
   - *Has it influenced who you speak to? Who you interact with? Where you get information? How you plan for healthcare visits?*

QUESTIONS ABOUT APP UTILITY AND PERCEIVED VALUE

1. Now that you've had a chance to play with the app, how do you feel about whether or not it was a good use of your time?

2. Would you recommend the App for healthcare transition? Why or why not?

QUESTIONS ABOUT PARTICIPATION IN VIRTUAL RESEARCH STUDY

1. What was your experience of being enrolled in a study virtually?

2. When it comes to participating in research, did you find the process of a virtual visit more helpful or less helpful? Why?
   Probe: What specifically did you find to be helpful? Not helpful?

Thank you for participating. Your opinions and input are very appreciated, and will help to make the App the best it can be for the youth who will be using it in the future.
