# Supplementary material for: An e-health transition intervention for youth with brain-based disabilities: Pilot and feasibility results from a Randomized Controlled Trial
Source: Health Care Transit. 2026 Jun 10;4:100144. doi: 10.1016/j.hctj.2026.100144 (PMC13273774; doi:10.1016/j.hctj.2026.100144)
Supplement: Supplementary material [file mmc5.pdf]

Supplemental File 5. Parent/Caregiver Qualitative Interview Guide.

CHILD-BRIGHT READYorNot™ Brain-Based Disabilities Trial
Parent/Caregiver Interview Guide

Interview Logistics

**Participant ID**	
**Interview Date** (month/day/year)	
**Interviewer**	
**Length of Interview** (minutes)	
**Additional Notes**	

Introduction

Hello, my name is [name] and I am [position] on the READYorNot™ Brain-Based Disabilities Trial. Thank you for agreeing to take part in this interview. As a reminder, we are conducting this study to see if, for youth who are 15-17 years old with a brain-based disability, it is better to use the MyREADY Transition App or not to use it. We want to know if using the App can improve healthcare transition experiences (moving from pediatric to adult health care). You have been invited to take part in this interview because your child is in the group who received the App as part of this Trial.

The purpose of this interview is to ask you about the App, how you may have used it with your child, and how you feel about its usefulness for healthcare transition. There are no right or wrong answers. The interview today will last about 30 minutes and will be audio-taped if you are okay with that. Your participation is completely voluntary, and we can stop the interview at any time. The information you share here will be kept private. Researchers involved in the READYorNot™ Brain-Based Disabilities Trial will be able to read what you tell me, but they will not see your name attached. Do you have any questions?

Can I confirm if you are still interested to participate in this interview ☐ Yes ☐ No

If it is acceptable to you, I would like to record our conversation today. This will allow me to engage in conversation with you without having to take notes. Please note that once recordings are transcribed, they are deleted.

Would you be ok with recording the conversation today? ☐ Yes ☐ No
(If not: Ok, I will take written notes instead to document our conversation).

INTERVIEW QUESTIONS

1. In general, what was your experience as a parent/caregiver with the study and with the study intervention (i.e., the App)?
   Probes:
   - *What was your experience with the study being conducted virtually? With App set-up occurring virtually?*

2. Can you tell me a little bit about how your child used the App? How did it go while they were in the study?
   Probes:
   - *Did your child have issues (e.g., technological) using the App?*
   - *Do you think your child enjoyed using the App?*

3. What was your involvement with the intervention (i.e., your engagement with the App and support of your child using the App)?
   Probes:
   - *Did you help with setting up the App? Troubleshooting technological issues?*
   - *Did your child have questions for you because of using the App? Did you ask your child anything about the App?*
   - *Did you work together with your child on any parts of the App? What parts?*

4. In general, what do you think about the intervention (i.e., App) of this study? What do you think of what you saw in the App? [show visuals if parent/caregiver unfamiliar with App and content]
   Probes:
   - *Do you think the App seems useful for healthcare transition? Why or why not?*
   - *Did you notice the App influenced ways in which your child interacted differently with you? With their healthcare providers? With their siblings? With their friends? (e.g., How they made decisions? Planned for appointments? Talked about their healthcare? Set goals?)*
   - *Do you feel the App was appropriate for your child? (e.g., content, accessibility, language level)*
   - *Do you have any suggestions for us that may help improve the App in the future?*
   - *Is there any other information you would like to see included in the App?*

5. Would you recommend the App to other families of children with a disability who are preparing for healthcare transition? Why or why not?

Thank you for participating. Your opinions and input are very appreciated, and will help to make the App the best it can be for the youth who will be using it in the future.
