# Supplementary material for: An e-health transition intervention for youth with brain-based disabilities: Pilot and feasibility results from a Randomized Controlled Trial
Source: Health Care Transit. 2026 Jun 10;4:100144. doi: 10.1016/j.hctj.2026.100144 (PMC13273774; doi:10.1016/j.hctj.2026.100144)
Supplement: Supplementary material [file mmc6.pdf]

Supplemental File 6. Focus Group with Research Assistants Summary.
CHILD-BRIGHT READYorNot™ Brain-Based Disabilities Trial

Purpose:
After data collection for the randomised controlled trial (RCT) concluded, a focus group was conducted with research assistant (RA) staff who were involved in recruiting youth participants for the RCT. The purpose of the focus group with RAs was to learn more about experiences related to the processes, resources, and management of the study, and about how the COVID-19 pandemic may have impacted each of these things. The focus group results will enhance our pilot and feasibility exploration and reporting.

Participants:
Feedback was received from a total of 6 RAs who were on staff at one of the participating study sites, with representation of all 4 RCT regions (Ontario, Alberta, the Maritimes, and Quebec). The focus group was facilitated by a graduate trainee on the project. The recorded focus group session was 80 minutes in length. Three RAs participated in the focus group directly and were invited to review the summary, giving them the opportunity to add to or expand on the ideas discussed. RAs who did not attend the focus group were invited to provide asynchronous feedback on the summary (and feedback was received from 3 additional RAs).

Questions considered:

1. We are interested in learning about the **processes** involved in finding study participants, enrolling them in the study and collecting data. For the following list: Can you tell me what challenges you faced? Can you tell me about your tips/strategies/successes?
   - *Screening*
   - *Recruitment*
   - *Retention*
   - *Adherence*
   - *Data collection*

2. We are interested in learning about the **resources** required to conduct the study. For the following list: Can you tell me what challenges you faced? Can you tell me about your tips/strategies/successes?
   - *Timing and duration of study visits*
   - *Equipment availability*
   - *General scope and workload*

3. We are interested in learning about the day-to-day **management** of the study. For the following list: Can you tell me what challenges you faced? Can you tell me about your tips/strategies/successes?
   - *Study personnel*
   - *Ethics and administrative management*
   - *Training/communication*
   - *Tracking and data entry software*

Feedback Received (challenges, tips, strategies, successes):

PROCESS

- *Screening* for eligibility was not difficult once a candidate was recruited. The initial screening was done by telephone rather than Zoom which made it a bit tougher to build rapport when you could not see each other, and sometimes they did not respond to the phone call right away. *S*creening challenges were related to the transition readiness screening criteria (TRANSITION-Q conversion score ≥ 41).
  - Some of the items on the TRANSITION-Q felt out of range for younger youth. For example, they may have been too young to have a license to drive to appointments. Tip: Explaining that not everyone in the study is expected to be doing all these things, but that we need a standardized way to measure readiness using the same questions for everyone.
  - Sometimes youth were disappointed when they did not quite meet the TRANSITION-Q cut-off for eligibility. Tip: If they were close to cut-off and they were interested, offering to retry the questions again in a month or two, to see if they would become eligible. However, since they knew the items already, they could embellish answers the next time to increase their score.
- *Recruitment* was the most challenging aspect overall. Physical distancing restrictions and staff re-deployment due to the COVID-19 pandemic were factors that contributed to recruitment challenges and made it even more important to find creative strategies besides traditional clinic recruitment, such as recruiting through social media and community organizations. Youth with brain-based disabilities who would be eligible may no longer be followed in a hospital pediatric clinic but rather by their family doctor.
  - With restrictions due to the COVID-19 pandemic and schools being delivered primarily online, some parents were cautious about the amount of added screentime, and so may have been less keen for youth to participate.
  - When physical distancing restrictions were lifted, and we were able to meet in person, face-to-face recruitment conversations were more successful than virtual. When it was possible, the ability to go and meet youth at their clinic appointment was helpful, and the physical presence of the RA in clinic may also help remind clinicians to ask youth about the study.
  - Screening and extra protocols due to COVID-19 made clinics busier, so it was harder to get the attention of the clinic staff to help recruit in clinic.
  - When clinical staff were re-deployed to other areas as a COVID-19 measure, it reduced capacity to help recruit for research. It was understandable given the priorities at the time and the reality of the situation. Research was something extra or paused for a time, as a lower priority. We had to respect the priorities of clinics and not add more pressure on the system. One site reported that staff was rapidly back to the office to work and see patients; however, clinics took a longer time to come back to regular caseload, mostly doing remote visits with their doctor. Staff were generally busier due to pandemic, so we had to be extra careful with how to approach clinicians and clinic staff at that time.
  - An efficiency for future research is to seek ethics approval from the outset for multiple recruitment strategies. For example, sharing recruitment posters in the community and/or in family doctor offices.
  - The length of time required to receive ethics and have contracts in place resulted in reduced recruitment period at some sites. [Delays were often institutionally-driven and were further amplified by COVID-19 related projects being given higher priority in contracts and ethics offices].
  - Youth were either interested or not at all interested, no gray area. Some felt they needed preparation for healthcare transition, others were less inclined to participate in a study that reminds them of their condition.

  - Invest in direct personal connections and building these connections over time. For example, scheduling a one-to-one meeting or presenting at a team meeting. This investment can help to receive buy-in and raise awareness about the study generally and ultimately help with recruitment. Receiving buy-in from the department head can help prioritize the study. Administrative staff are great recruitment contacts as they may be more responsive and available than clinicians. At some sites these connections were made at the start of the project but because of approval delays, too much time had passed and connections were not re-established.
  - When recruitment was ending, people rallied together and were motivated by having an impending deadline. Setting deadlines is a helpful strategy for future.
- *Retention* rate overall was respectable and in line with what we see in other research projects based on the experiences of RAs. Certain times of year are busier than others and depending on when the follow-up landed, this may have contributed to lack of follow through with 6-month questionnaires. Some strategies to optimize retention were discussed:
  - Better incentives may help motivate youth to follow through. Ongoing communication with participants during the 6-month follow-up period may be another motivator to keep participants engaged to the end. We sent one family newsletter during the study.
  - Frustration with the App and technical issues, or not using the App may have contributed to lack of follow through to the 6-month visit for participants in the intervention group.
  - Using various strategies (e.g., phone, email, text messages) to contact people for follow-up was most helpful to optimize retention. The use of certain strategies may be limited by a site's policies and ethics approvals. Sending the link to 6-Month questionnaires by email, then being understanding and offering to schedule a phone appointment to complete these questionnaires was quite successful when they did not already complete it independently.
- *Adherence.* After the initial visit, RAs felt removed and did not know what participants were doing with the App, in terms of adherence or requests for technical support.
  - The Getting Started video and How-To videos (on the App support website) were very well done. Everything was clear, and youth were able to follow along with the RA in getting set up to use the App.
  - RAs were to receive notifications when it was time to send encouragement emails at various App progress points for participants in the intervention group, but that did not happen consistently. RAs would like to have received reports about App usage more frequently. [The encouragement emails were designed as a strategy to increase uptake of the App in a standardized way. Messages were not sent consistently due to gaps in the project manager's access the App's CMS tracking. Ideally these encouragement messages would be pushed directly by the App in a future App version].
  - The ability to use the App on multiple devices might have helped with adherence. Sometimes the participant had the App on a device that was shared with other members of the family. For example, one participant had a shared device with a sibling. At one point we had considered providing participants with a tablet to use for the study.
  - Some youth with or a short attention span (for example, those with FASD and/or ASD) who had technical issues/glitches/freezing/losing progress became frustrated which made it very difficult to get them to go back and try it again. For example, because using the App was hard in the beginning, the youth did not want to try the App again and the parents did not choose to battle about participating in the study when they were dealing with other behaviour and regulation issues in day-to-day life.
  - Ideally resources should be implemented within the App, if they need to search somewhere else or on a separate document, this will not be prioritized, too much effort or they will forget they have the information. Technical glitches will always happen so it would be ideal

also to offer some of the App's resources in another format to get some information to youth who really will not try again. For example, the resources could be offered as a pdf brochure format.

- *Data collection* went smoothly, and RAs already had familiarity with REDCap.
  - One RA reported initial confusion about participant ID numbering protocol for their site, which was resolved with help from the central coordination.
  - RAs typically met with both the parent and youth to start, then the parent would go off nearby and complete their data collection forms independently. This approach worked well to have the parent nearby, because sometimes youth would ask their parent a question or confirm a detail. One RA noted that the PEDS-QL questions may be awkward to answer with a parent nearby. For example, the youth may want to answer questions about their interactions with their peers and/or their fears with their parents present.
  - Some youth self-completed the data collection forms. When possible, RAs recommend the strategy to go through the data collection forms together with the youth by sharing the screen and reading questions aloud. This approach helps to build a bit of a relationship, where there could be opportunities for joking and relating experiences with each other to make the interaction feel more personal. The COPM measure was helpful to learn a bit about them and their personal interests and the connection was stronger after that. RAs tried to recall some things that they had talked about at the first visit to make their next session more personal. For example, "Last time we talked, you were applying to university. How are things going with that?"
  - REDCap was user-friendly and sending the link by email for questionnaires was easy to do for the most part. Glitches in REDCap were found and resolved (e.g., parent survey link sending as if they had already done it; baseline COPM text not piping in the 6-month form).
  - The COVID-19 pandemic made visits "online friendly". Youth were already accustomed to doing things virtually since they were doing schooling online. Using Zoom made it easy for RAs to share their screen to see videos and questionnaires together. Further, the RAs found that "meeting youth where they were at" online was helpful.

RESOURCES

- *Timing and duration of study visits.* Visits were mostly done using Zoom and were scheduled at the convenience of families, so RAs needed to be flexible to work in the evening and/or on weekends. The duration of most visits was 1-1.75 hours. Some younger youth took longer to come up with things for the COPM. One RA talked about appointments being excessive in a duration of 2-3 hours. RAs offered to break up the session, give a short break if needed, or provide the introduction to the App in a separate session that would be scheduled soon after the initial visit.
- *Equipment availability* did not seem to be an issue as everyone had a device, or computer available. As per the screening, they knew they needed to have access to a device, whether it was their own or shared with other family members, to be eligible to participate. There was one issue mentioned where a participant shared a device with their sibling and the sibling accidentally deleted the App. RAs shared that it would be helpful to have devices for participants just in case.
- *General scope and workload.* The RA workload was not unmanageable, however scheduling time outside regular working hours could be tricky. One RA commented that with social media recruitment, you cast a wide net, but may only receive meagre response and not yield many participants. For example, they contacted hundreds of organizations and groups asking them to

share the recruitment ad in e-newsletters or on social media, and the uptake was modest with few responses.

MANAGEMENT

- *Study personnel* remained consistent with no turnover at any of their sites, although there was a change in local PI at one site, with minimal impact as this change occurred at the end of the study. One RA reported being re-deployed to do COVID-19 screening in the hospital for a short time. Another RA commented about being external to the hospital and clinics, which made it challenging to connect with or get a response from clinicians. When the RA is external and not in the circle of care, there are additional steps needed for recruitment. For example, someone in the circle of care must be the first point of contact with participants. In this case, the PI and RAs needed to develop rapport with the clinicians such as by providing a short presentation about the study in a meeting or research rounds with clinicians.
- *Ethics and administrative management.* Everyone agreed that the wait and timing for both contracts and ethics can be long and may be more so at certain times of year. In one region (Ontario) we were able to use a provincial streamlined system where a single Research Ethics Board provides ethics review and ongoing oversight for multiple research sites. The system works quite well, although one RA reported reluctance from their site, who still required a parallel local application initially which led to double the amount of work. In this case, after some advocacy from the RA and local PI, a positive outcome was that the site does now accept the streamlined approval. The Quebec region also has a provincial streamlined process, where if more than one site is involved in the same study, one participating site will take lead as the 'main coordinating center' and its REB takes the role to review the study (ethics and scientific) and provides first approval for all sites. At each site, the local REB needs only to perform a short review of documents to make sure it respects local procedure and legal documents are completed. This is to avoid duplication of review of the same study by all REBs. However, when the main coordinating site has problems or delays with their ethics, then all other sites are affected because they cannot proceed until main site obtains approval first. Since Quebec is a bilingual province, all patient facing documents and the App intervention needed to be available in French to have approval as well (an additional requirement that other provinces do not have). Another RA reported a cumbersome process in another region (Maritimes) and had to apply separately to four different boards, at two recruiting sites and two universities. The submission of multiple ethics applications was time-consuming and tricky to keep them all updated. RAs shared that keeping things like consent forms standardized as much as possible helps to keep things organized. The RAs found it helpful that the central research coordinator provided a summary of changes with new document files showing all track changes.
- *Training/communication*. All RAs reported feeling very well prepared and supported both by central coordination staff and by their local PI. They understood what was expected, what to do and were happy with the support with quick and detailed responses from central coordination whenever they had a question. They thought the e-learning modules for training were very well done and engaging compared to other training they had done. The mock visits and debrief sessions made them feel comfortable before they started recruiting. RAs liked that they could refresh on a topic at any time by re-watching the YouTube videos that were part of the training e-learning modules. The protocol was also written in a way that was easy to follow. Central coordinators were available for ongoing questions and offered re-training to RAs whose approval and recruitment start was far from their initial training.

- *Tracking and data entry software.* RAs used the tools and resources provided in the REDCap File Repository. The Excel templates for tracking that were broken down into multiple steps also served as a good checklist. Having the REDCap auto reminders was helpful to make sure a follow-up visit was not forgotten. Health card numbers were collected separately from REDCap. One RA had the parent repeat the numbers to double check and verify that the health card number was recorded correctly. Another RA reported that the site required the health card number was in writing, signed and mailed back rather than given verbally, which was an added step that may have contributed to fewer people providing it at that site.
