# Supplementary material for: An e-health transition intervention for youth with brain-based disabilities: Pilot and feasibility results from a Randomized Controlled Trial
Source: Health Care Transit. 2026 Jun 10;4:100144. doi: 10.1016/j.hctj.2026.100144 (PMC13273774; doi:10.1016/j.hctj.2026.100144)
Supplement: Supplementary material [file mmc7.pdf]

Supplemental File 7. Health Care Provider Electronic Survey Summary.
CHILD-BRIGHT READYorNot™ Brain-Based Disabilities Trial

Purpose:
After data collection for the randomised controlled trial (RCT) concluded, we conducted an electronic survey as optional research to enhance our pilot and feasibility exploration and reporting. The 19 participants invited to complete the electronic survey were health care providers (HCPs) who were involved in recruiting youth participants for the RCT in all four regions (11 in Ontario, 3 in Alberta, 3 in the Maritimes, and 2 in Quebec). The survey included two open-ended questions to learn about HCP experience recruiting youth during the COVID-19 pandemic and about their perspective on integrating the App intervention into practice.

Participants:
Feedback was received from 5 health care providers, with representation of one study region only (Ontario).

Questions considered:

1. **Please tell us about your experience recruiting youth during the COVID-19 pandemic. Were there particular challenges or successes you would like to share? We are particularly interested in how the shift from in person to virtual care may have impacted recruitment and if you have any tips or tricks that you found useful for reaching and enticing youth to participate in research.**
    - "Difficult not being in person, reviewing patient lists were helpful"
    - "Recruiting is challenging without a research assistant readily available on site even when patients are present in person. Our virtual model of care shifted a number of our appointments to an NP [nurse practitioner] provider which also impacted my ability to directly impact recruitment. From other studies, I think being able to communicate with participants via text/email makes a difference to recruiting in a virtual world!"
    - "I usually recruited clients at the end of our virtual appointment [name redacted]. While recruits were very excited to participate, they may have had virtual activity fatigue since school was also virtual. I think participating virtually in a study like this is a good idea since it actually allows for better focus as long as the recruits are not engaging in other activities such as school or healthcare appointments. I noticed that youth had a tendency to prioritize school over attending a virtual appointment, almost as if they had control to prioritize since it the decision was youth driven. When parents are involved as in the case of onsite appointments there seems to be shared responsibility and the youth are literally brought to clinic to engage. I think in a trial like this, dual participation, both parent and youth should be offered, if not already. The parents usually show great interest in transition material and I think using their support to leverage any type of learning or support is critical."
    - "During the pandemic we had mostly virtual visits in various clinics at the hospital. Firstly the frequency of visits were reduced as we had less efficiency, so I saw fewer adolescents. Secondly at the appointments the focus of youth and families often was on

pressing (Health) issues, less on develpmental processes like transition. The good thing of videocalls was that I could share screen and walk the youth (and their parents) through the flyer with information as well as showing a short recruitment video."

- "I was glad to recruit though with pandemic, so many additional tasks with remote work, and lack of a paper to hand to them it was definitely more challenging."

2. **Please tell us about your impressions of the MyREADY TransitionTM BBD App. For example, its value for your patients, and how you might see an eHealth intervention like this being integrated into clinical practice.**

- "Great resource"
- "I think an app like this could be helpful for a subset of patients, typically those with higher levels of function who are more likely to achieve a level of independence with self management. A challenge is keeping it up to date and relevant, both for new and long time users."
- "I think this app is very valuable as it is a teaching tool. I can see integrating it into clinical practice by offering it and having youth use it while they are waiting in the waiting room for their appointment or review it before our one to one transition virtual appointment that we are currently trialing in collaboration with an acute care pediatric hospital for our shared patients. Also, having youth review the app a week prior to clinic appointment so that we can have a meaningful discussion about transition at their routine appointment would be a great opportunity. Running another trial in this context may be worthy."
- "Youth are interested in the interactive, gaming elements of the app. The curriculum of reading and understanding transitions seems less interesting to youth, altough it did help raise awareness. The integration of such an eHealth intervention un clinical practice is of uptmost importance. Youth need to dvelp skills and knowledge which can onlt be done through practice in real life situations. Therefor a HCP needs to invite the youth to share their experiences with the app, check hat their goals are and attune the clinic visit to the goal (for example asking questions and meeiting a doctor on their own). The Transition_Q , amog other tools, can serves as a spingboard to start a conversation, set goals and practice a skill as they are relevnat to the person and family."
- "I believe, though blinded to whether it was used, I could detect more confidence presenting at appointments, willingness to engage and lead in meetings."
