# Supplementary material for: An e-health transition intervention for youth with brain-based disabilities: Pilot and feasibility results from a Randomized Controlled Trial
Source: Health Care Transit. 2026 Jun 10;4:100144. doi: 10.1016/j.hctj.2026.100144 (PMC13273774; doi:10.1016/j.hctj.2026.100144)
Supplement: Supplementary material [file mmc8.pdf]

Supplemental File 8. Scientific Feasibility (estimated initial treatment effects): Primary and Secondary Outcomes at 6-month follow-up

Outcome	Intervention (n=19)	Usual Care Control (n=24)	Mean difference* (95% CI) (Intervention - Control)
**Primary Outcomes**			
**TRAQ self-management; mean (SD)**	2.5 (0.7)	2.5 (0.9)	-0.16 (-0.56, 0.24)
**TRAQ self-advocacy; mean (SD)**	3.6 (0.6)	3.2 (0.8)	0.003 (-0.35, 0.36)
**Secondary Outcomes**			
**PedsQL™ (Overall); mean (SD)**	63.2 (18.3)	66.2 (18.1)	-2.05 (-9.44, 5.34)
Physical health summary	67.8 (27.2)	67.9 (29.4)	
Psychosocial health summary	60.9 (18.8)	65.2 (16.9)	
Emotional functioning subscale	57.9 (17.8)	58.5 (20.8)	
Social functioning subscale	64.7 (27.7)	72.7 (22.5)	
School functioning subscale	59.2 (18.8)	62.1 (20.2)	
**TRANSITION-Q; mean (SD)**	59.4 (13.2)	52.2 (19.6)	5.99 (-2.49, 14.47)
**COPM-P; mean (SD)**	6.0 (1.6)	6.7 (2.3)	-0.09 (-1.04, 0.85)
**COPM-S; mean (SD)**	6.3 (1.9)	7.3 (2.3)	-0.74 (-2.06, 0.59)
**MPOC Scores; mean (SD)**			
Enabling and partnership	4.5 (1.8)	4.4 (1.7)	
Providing general information	3.3 (1.7)	3.6 (1.8)	
Providing specific information	4.2 (2.1)	4.4 (1.6)	
Coordination and comprehensive care	4.6 (2.0)	4.7 (1.6)	
Respectful and supportive care	4.8 (1.6)	4.9 (1.5)	

*Mean difference estimates were obtained utilizing Analysis of covariance (ANCOVA) on imputed data. All analyses have been adjusted for baseline and region. Interaction terms were dropped from the final model since no interaction effects were observed between region and intervention.

SD=Standard deviation; TRAQ=Transition Readiness assessment Questionnaire; PedsQL™=Pediatric Quality of life; COPM-P=Canadian Occupational Performance Measure - Performance; COPM-S=Canadian Occupational Performance Measure - Satisfaction; NVS= Newest Vital Sign; MPOC=Measure of Process of Care.
