# Supplementary material for: An e-health transition intervention for youth with brain-based disabilities: Pilot and feasibility results from a Randomized Controlled Trial
Source: Health Care Transit. 2026 Jun 10;4:100144. doi: 10.1016/j.hctj.2026.100144 (PMC13273774; doi:10.1016/j.hctj.2026.100144)
Supplement: Supplementary material [file mmc9.pdf]

Supplemental File 9. Priorities that were most pressing or important to youth from the Canadian Occupational Performance Measure (COPM).

COPM category	COPM subcategory	Intervention (n=19)	Usual Care Control (n=24)
Self Care; n (%)	Managing physical and mental health (medication, appointments, feelings, being at my best)	5 (26.3)	12 (50.0)
	Mobility (getting around)	2 (10.5)	4 (16.7)
	Transportation (public transit, driving, travel)	8 (42.1)	2 (8.3)
	Dressing (getting ready)	3 (15.8)	3 (12.5)
	Eating	2 (10.5)	2 (8.3)
	Sleeping	3 (15.8)	2 (8.3)
	Communication (expressing yourself)	2 (10.5)	2 (8.3)
	Hygiene (face, hair, teeth)	2 (10.5)	3 (12.5)
	Bathing (shower)	0 (0.0)	2 (8.3)
	Toileting	0 (0.0)	1 (4.2)
	Community management (living independently, getting groceries, navigating)	1 (5.3)	0 (0.0)
Leisure; n (%)	Active leisure (fitness, healthy lifestyle, sports, dance)	9 (47.4)	15 (62.5)
	Socialization (relationships)	6 (31.6)	11 (45.8)
	Quiet leisure (reading, art, creative writing, music, gaming)	5 (26.3)	11 (45.8)
Productivity; n (%)	School (homework, studying)	14 (73.7)	14 (58.3)
	Household management (garbage, dishes, laundry, cooking, cleaning)	9 (47.4)	11 (45.8)
	Work/volunteering (productivity)	9 (47.4)	5 (20.8)
		80 goals overall	100 goals overall
